# The Role of Cellular Senescence and SASP in the Pathogenesis of Atherosclerosis and the Therapeutic Potential of Senolytic Strategies in Cardiovascular Diseases

**DOI:** 10.3390/biomedicines14020331

**Published:** 2026-01-31

**Authors:** Zuzanna Krupa, Joanna Wrona, Marta Zawadzka, Julia Rydzek, Julia Lizon, Paulina Kalemba, Konrad Kochman, Paweł Iwaszkiewicz, Robert Iwanowski, Sławomir Woźniak

**Affiliations:** 1 Student Scientific Society Anatomia-Klinika-Nauka, Division of Anatomy, Department of Human Morphology and Embryology, Wroclaw Medical University, 50-367 Wroclaw, Poland; zuzanna.krupa@student.umw.edu.pl (Z.K.); joanna.wrona@student.umw.edu.pl (J.W.); julia.rydzek@student.umw.edu.pl (J.R.); julia.lizon@student.umw.edu.pl (J.L.); paulina.kalemba@student.umw.edu.pl (P.K.); 2 J. Gromkowski Voivodship Specialist Hospital in Wroclaw, 51-149 Wroclaw, Poland; konrad.kochman@interia.pl; 3 T. Marciniak Lower Silesian Specialist Hospital in Wroclaw, 50-065 Wroclaw, Poland; pawel.iwaszkiewicz20@gmail.com; 4 Regional Health Centre in Lubin, 59-300 Lubin, Poland; robcio20002016@gmail.com; 5 Division of Anatomy, Department of Human Morphology and Embryology, Wroclaw Medical University, 50-367 Wroclaw, Poland; slawomir.wozniak@umw.edu.pl

**Keywords:** cellular senescence, senescence-associated secretory phenotype (SASP), atherosclerosis, cardiovascular diseases, senolytics, senomorphics

## Abstract

Cellular senescence is a permanent cell cycle arrest that plays a critical role in the development and pathogenesis of age-related diseases. This paper aims to present the biological mechanisms of cellular senescence and the role of the senescence-associated secretory phenotype (SASP) in the pathogenesis of atherosclerosis, as well as to discuss therapeutic strategies targeting senescent cells in cardiovascular diseases. Different types of cellular senescence are described, including replicative, stress-induced, and oncogene-induced senescence, along with the composition and regulation of SASP and its impact on chronic inflammation, endothelial dysfunction, vascular remodeling, and plaque destabilization. The involvement of senescent endothelial cells, vascular smooth muscle cells, and macrophages in the initiation and progression of atherosclerosis is also discussed. The paper reviews current research on senolytic and senomorphic therapies and highlights emerging approaches such as immunosenolytic and epigenetic interventions. The therapeutic potential of these strategies in reducing chronic vascular inflammation and improving plaque stability, as well as their limitations and challenges in clinical application, is emphasized.

## 1. Introduction

Atherosclerosis is a chronic inflammatory disease of the arterial walls and a major cause of cardiovascular diseases (CVD), including acute myocardial infarction and ischemic stroke [1,2]. Numerous modifiable and non-modifiable risk factors contribute to its development, such as smoking, diabetes, hypertension, hyperlipidemia, and low physical activity [3]. Aging is recognized as one of the strongest risk factors for atherosclerosis [4]. A key component of aging is cellular senescence, which through the Senescence-Associated Secretory Phenotype (SASP) can drive chronic inflammation.

The cell cycle of senescent cells is irreversibly arrested, primarily mediated by upregulation of cell cycle inhibitors p16, p21, and p53 [5]. Senescent cells also exhibit cytoplasmic enlargement and a flattened, irregular morphology [2]. These cells display a characteristic proinflammatory phenotype known as SASP, which induces the secretion of chemokines and cytokines (such as IL-1β, IL-6, and HMGB-1), promoting local and systemic inflammation, immune activation, tissue damage, and dysfunction and are considered risk factors for cardiovascular diseases [6,7]. SASP also enables senescent cells to communicate with neighboring cells and the microenvironment, promoting paracrine senescence. Senescent cells are more resistant to both intrinsic and extrinsic pro-apoptotic stimuli [8].

Senescence of vascular smooth muscle cells (VSMCs) is closely associated with the development of atherosclerosis and plaque instability. Senescent VSMCs promote plaque calcification and enlargement of the necrotic core via SASP. Additionally, senescent immune cells in the vascular wall contribute to atherogenesis. Senescent T lymphocytes secrete numerous proinflammatory factors, such as TNF and osteopontin, displaying SASP-like features [6].

Therapies targeting senescent cells, including senolytic drugs, selectively induce the elimination of these cells and can delay or mitigate many age-related diseases, including CVD [9].

Senolytics represent a novel class of agents that exploit differences between senescent and non-senescent cells to stimulate apoptosis of the former [10]. They act, for example, by inhibiting anti-apoptotic BCL-2 pathways (e.g., ABT263) [11] or tyrosine kinases (dasatinib) [12]. Some cardiovascular drugs, such as digoxin, have also been shown to possess senolytic properties, although their use in CVD may be limited due to potential adverse effects [13]. As SASP is a major mediator of tissue damage during aging, senomorphic therapies are also being developed, which modulate SASP without eliminating senescent cells. These therapies have the potential to achieve many of the beneficial effects of senolytics with reduced toxicity [10]. Senolytic drugs represent a promising therapeutic strategy, opening new avenues for the treatment and prevention of age-related CVD [14].

## 2. Materials and Methods

This article is a narrative review based on a structured literature search focusing on cellular senescence, the senescence-associated secretory phenotype (SASP), and their roles in atherosclerosis and cardiovascular diseases.

Literature searches were performed in PubMed, Scopus, and Web of Science databases and covered publications from January 2005 to June 2025. The following keywords were used in various combinations with Boolean operators (AND, OR): “cellular senescence”, “senescence-associated secretory phenotype”, “SASP”, “atherosclerosis”, “cardiovascular diseases”, “senolytics”, “senomorphics”, “endothelial dysfunction”, and “vascular smooth muscle cells”.

The initial search identified approximately 320 records. After removal of duplicates, titles and abstracts were screened for relevance. Studies were included if they addressed mechanisms of cellular senescence or SASP, their involvement in atherosclerosis or cardiovascular diseases, or senescence-targeted therapeutic strategies. Only English-language publications were considered, while editorials, conference abstracts, and studies not directly related to cardiovascular pathology were excluded.

Following full-text assessment, 84 publications were included, comprising mainly original experimental and translational studies, supported by selected review articles for conceptual background. As this was a narrative rather than a systematic review, the selection process was descriptive and no PRISMA flowchart was applied. The included literature was analyzed narratively to summarize current knowledge on senescence mechanisms, the role of SASP in atherosclerosis, and emerging therapeutic strategies in cardiovascular diseases.

## 3. Biology and Mechanisms of Cellular Senescence

Cellular aging leads to permanent and irreversible growth arrest. Senescent cells accumulate and contribute to adverse tissue remodeling, organismal aging, and the pathogenesis of age-related diseases [15]. One type of senescence is replicative cellular senescence, which is a necessary physiological process but is more widely recognized for its role in organismal aging, cancer, and other pathologies [16]. It is induced by telomere shortening, which contributes to the loss of regenerative capacity in organs [17]. Telomeres are the ends of chromosomes that shorten each cycle of DNA replication. When they become critically short, they trigger proliferation arrest, referred to as replicative senescence [18]. Studies have shown that in stress-induced cellular senescence, primarily caused by DNA-damaging external factors, damage accumulates mainly at telomeres, even when telomeres are long and telomerase activity is normal [19]. This suggests that telomere deterioration is not solely due to shortening but also to DNA damage within telomeres [20]. Two main mechanisms can counteract telomere shortening: the enzyme telomerase, which synthesizes new telomeric sequences, and DNA repair mechanisms collectively referred to as Alternative Lengthening of Telomeres (ALT). However, in healthy human cells, these mechanisms cannot fully compensate for telomere shortening. Strong activation of these mechanisms is observed in cancer cells [16].

Classic features of cells undergoing replicative senescence include transcriptomic changes, such as increased activity of senescence-associated β-galactosidase (SA-β-Gal) and elevated levels of p16 and p21 [21]. SA-β-Gal originates from lysosomes and is a lysosomal β-D-galactosidase. Under suboptimal pH (6.0), β-Gal activity is undetectable in most non-senescent cells but becomes detectable in senescent cells. This occurs because senescent cells have an increased number of lysosomes and elevated activity of lysosomal enzymes, including β-Gal. However, detection of β-Gal activity at pH 6.0 is not specific for senescent cells, and despite its utility, it cannot be used alone as a definitive marker for identifying senescent cells [22].

Major tissue-damaging factors, such as oxidative stress, DNA damage, mitochondrial dysfunction, and metabolic disturbances, can trigger cellular senescence [23]. Stress-induced premature senescence (SIPS) is a proliferation arrest triggered by stress factors such as oxidative stress, radiation, toxins, or overexpression of proto-oncogenes. These cells exhibit a senescent phenotype (SA-β-Gal activity, mitochondrial and epigenetic changes), resistance to apoptosis, and can appear independently of telomere length [24]. One key difference between replicative and stress-induced senescence is that stress-induced SASP [20]. Senescent endothelial cells (ECs) impair endothelial integrity and contribute to diseases such as atherosclerosis. Oxidative stress is a major driver of endothelial aging, with hydrogen peroxide (H_2_O_2_) and high glucose being key stressors. Exposure to H_2_O_2_ increases the number of cells positive for SA-β-gal and induces typical morphological changes associated with senescence, such as enlarged and flattened cell shape. It also increases the expression of senescence markers p16, p21, and p53 [25]. p53 is a transcription factor critical for cellular stress responses. It regulates cell cycle arrest, DNA repair, senescence, and apoptosis. In cellular senescence, p53 can be activated in both DNA damage-dependent and independent manners. Key pathways regulating cellular senescence include the tumor suppressor pathways p53/p21^Cip1^ and p16^INK4A^/Rb. While the p53 pathway mainly initiates senescence, p16 primarily maintains it. After induction of senescence, p53 levels decline, whereas p16 levels remain persistently high. The p16 pathway involves various Rb family proteins, with p130 playing a central role in stabilizing cell cycle arrest during senescence progression in human cells. High p16 activity prevents p53 downregulation from reversing proliferation arrest, and the combined action of p53 and pRb induces senescence in almost all cells [26]. The p21 pathway belongs to the Cip/Kip family of cyclin-dependent kinase inhibitors. It acts primarily as a cell cycle inhibitor, senescence inducer, and tumor suppressor. By inhibiting cyclin-dependent kinases, it arrests cell cycle progression at the G1/S and G2/M transitions. p21 also participates in apoptosis, transcription, DNA repair, and cell migration. It has become a central focus in cellular senescence research, with p21^high^ cells forming a distinct senescent population separate from p16-associated senescent cells, highlighting its specific role in senescence [27].

Oncogene-induced senescence (OIS) is an irreversible proliferation arrest triggered by oncogene activation. It is a biologically significant response that limits malignant transformation [28]. OIS cells exhibit high p16 expression, and reduced p16 levels may allow escape from senescence and re-entry into the cell cycle [29]. Experimental in vitro and in vivo studies in human melanocytic nevi show OIS as a barrier to tumorigenesis. Up to 80% of these nevi contain the BRAFV600E mutation. Mutant melanocytes remain growth-arrested, with elevated p16 expression and positive SA-β-Gal staining. These nevi are benign, stable over long periods, and represent an in vivo example of OIS [28]. In human endothelial-derived mesenchymal stem cells, senescence is the primary response to oncogene expression, with OIS induction mediated by MEK/ERK, PI3K/AKT, p53/p21^WAF/CIP^/Rb, and p38/p16^INK4a^/Rb signaling pathways [30].

One model of cellular aging is the heterochromatin loss hypothesis, which proposes that heterochromatin degradation leads to changes in nuclear structure and gene expression in these regions, contributing to senescence. Loss of transcriptional silencing associated with heterochromatin breakdown occurs during aging in all eukaryotic organisms, and evidence suggests that accelerating or reversing this process may influence lifespan, either shortening or extending it [31]. Aging in organisms is associated with an imbalance between activating and repressive histone modifications, such as H3K4me3, which activates gene transcription by RNA polymerase II, and H3K27me3, which represses gene expression. Analyses (Western blotting, immunofluorescence, and genome profiling) show that this imbalance leads to gene expression changes characteristic of the senescent cell phenotype [32].

An important feature of cellular aging is mitochondrial dysfunction, which affects not only growth arrest but also SASP development and resistance to cell death. Mitochondrial changes in senescence include alterations in mass, dynamics, structure, and function: decreased NAD^+^ and ATP levels, accumulation of metabolites (TCA, DAMPs, and reactive oxygen species (ROS)) and reduced oxidative phosphorylation (OXPHOS). Senescence also impairs mitochondrial dynamics, leading to elongation and hypertrophy. This is associated with decreased expression of FIS1, a protein involved in mitochondrial fission through DRP1 recruitment, whereas FIS1 overexpression prevents elongation and reverses the senescent phenotype [33]. The major types and molecular contexts of cellular senescence discussed in this section are summarized in Table 1.

## 4. SASP and Mechanisms of Atherosclerosis Pathogenesis

SASP represents a complex and highly regulated secretory program that emerges in senescent cells undergoing irreversible cell cycle arrest. While cellular senescence initially functions as an essential tumor-suppressive and tissue-protective mechanism by halting the proliferation of damaged or stressed cells, the long-term persistence and accumulation of senescent cells leads to sustained SASP production, which profoundly disrupts tissue homeostasis. Accordingly, the senescence-associated secretory phenotype exhibits a clear functional duality, with beneficial effects during acute activation and deleterious consequences when chronically sustained (see Table 2). Biologically, SASP displays a dual nature: in the short term, it promotes wound healing, extracellular matrix remodeling, and immune-mediated clearance of senescent cells, whereas chronic SASP activity drives persistent inflammation, tissue degeneration, and age-related diseases [6,34].

The molecular composition of SASP is highly heterogeneous and includes pro-inflammatory cytokines such as interleukin-6 (IL-6) and interleukin-1β (IL-1β), a broad spectrum of chemokines (e.g., CCL2, CXCL1), matrix metalloproteinases (MMP-2, MMP-9), growth factors (VEGF, GM-CSF, TGF-β), ROS, and extracellular vesicles containing bioactive lipids, proteins, and microRNAs [6]. The exact SASP profile depends on senescence-inducing stimuli, including DNA damage, oxidative stress, telomere attrition, lipid overload, mitochondrial dysfunction, and viral or metabolic stress [6,34].

At the regulatory level, SASP production is controlled by multiple intertwined signaling pathways. NF-κB acts as the central transcriptional hub responsible for the induction of inflammatory cytokines and chemokines. The mechanistic Target Of Rapamycin (mTOR) pathway sustains SASP by promoting protein synthesis, metabolic reprogramming, and increased secretory capacity of senescent cells. GATA4 serves as a molecular link between DNA damage response signaling and SASP activation by stimulating NF-κB–dependent transcription. In addition, p38 MAPK signaling and the cGAS–STING pathway play crucial roles in amplifying SASP, particularly in response to cytoplasmic chromatin fragments and mitochondrial DNA leakage [6]. These pathways establish feed-forward loops that stabilize SASP expression and contribute to the long-term survival and immune resistance of senescent cells (a phenomenon described as a major barrier to efficient senescent cell clearance).

Through chronic SASP secretion, senescent cells actively reshape the tissue microenvironment. Elevated levels of cytokines and chemokines promote continuous immune cell recruitment and activation, while MMP-mediated degradation of extracellular matrix components alters tissue structure, elasticity, and mechanical stability. ROS generated by senescent cells exacerbate local oxidative stress, further damaging neighboring cells and enhancing inflammatory signaling [35]. Importantly, SASP can induce paracrine senescence in adjacent healthy cells, thereby expanding the senescent cell population and reinforcing a self-sustaining inflammatory network. As a result, SASP is a central driver of chronic low-grade inflammation, commonly referred to as inflammaging [6,35,36].

In the vascular system, the pathogenic consequences of cellular senescence and SASP are particularly evident in the development and progression of atherosclerosis. Senescent endothelial cells exhibit marked dysfunction characterized by increased oxidative stress, enhanced endothelial permeability, and reduced nitric oxide bioavailability. These changes impair vasodilatory capacity and promote endothelial activation, leading to increased expression of adhesion molecules such as ICAM-1 and VCAM-1. Consequently, monocyte adhesion and transmigration into the arterial intima are facilitated, accelerating the early stages of atherogenesis [34].

VSMCs undergoing senescence contribute to plaque progression through multiple mechanisms. Senescent VSMCs shift from a contractile to a synthetic and pro-inflammatory phenotype, accompanied by reduced collagen synthesis and impaired extracellular matrix organization. SASP derived from these cells enhances MMP activity, promotes vascular calcification, and weakens the fibrous cap that stabilizes atherosclerotic plaques [37]. Moreover, chronic SASP exposure disrupts VSMC survival and repair functions [6,38].

Macrophage senescence further accelerates plaque instability by impairing efferocytosis, altering immunometabolic pathways, and sustaining inflammatory cytokine production. Senescent macrophages show diminished ability to clear apoptotic cells, contributing to lipid accumulation, foam cell formation, and expansion of the necrotic core. SASP-associated proteases, particularly MMP-2 and MMP-9, degrade collagen and elastin within the fibrous cap, directly increasing the risk of plaque rupture and acute cardiovascular events [6,34,37,39].

Collectively, the accumulation of senescent endothelial cells, VSMCs, and macrophages, together with persistent SASP signaling, drives the initiation, progression, and destabilization of atherosclerotic plaques. These insights provide strong mechanistic support for the concept that senescence and SASP are not merely bystanders but active drivers of vascular pathology. Consequently, therapeutic strategies aimed at eliminating senescent cells (senolytics) or selectively suppressing harmful SASP components (senomorphics) have emerged as promising approaches to mitigate chronic vascular inflammation and reduce atherosclerotic disease burden [34,36,39].

## 5. Senolytic and Senomorphic Therapies in Atherosclerosis and Cardiovascular Diseases

### 5.1. Senolytic Therapies

Senolytics are a class of drugs designed to selectively eliminate senescent cells, which accumulate with aging and in chronic diseases such as atherosclerosis [6,40,41]. Cellular senescence is characterized by irreversible cell-cycle arrest, resistance to apoptosis, and acquisition of a SASP. In the vascular wall, senescent endothelial cells, VSMCs, and macrophages contribute to endothelial dysfunction, chronic inflammation, plaque instability, and impaired vascular repair [6,42]. The main mechanism of senolytic drugs is the disruption of senescence-associated anti-apoptotic pathways (SCAPs). Senescent cells increase the activity of pro-survival pathways that protect them from apoptosis, including BCL-2/BCL-xL, PI3K/AKT, p53/p21, and HSP90-dependent pathways, allowing them to resist programmed cell death [6,43]. Senolytics target these pathways, which selectively induce apoptosis in senescent cells without affecting normal cells [44]. Reduction in senescent cells results in a significant reduction in SASP factors, leading to a reduction in chronic vascular inflammation, oxidative stress, and endothelial dysfunction [45].

The combination of dasatinib and quercetin (D+Q) is one of the most extensively studied senolytic regimens. Dasatinib, a tyrosine kinase inhibitor, and quercetin, a natural flavonoid, act synergistically by targeting complementary SCAPs. This combination has been shown to eliminate multiple senescent cell types relevant to atherosclerosis [12,46]. Fisetin, another plant-derived flavonoid, exhibits potent senolytic activity and is considered one of the most effective naturally occurring senolytics identified. It targets anti-apoptotic signaling and reduces senescent burden with a favorable safety profile in preclinical models [47]. Navitoclax (ABT-263), an inhibitor of BCL-2 and BCL-xL, demonstrates senolytic efficacy, restraining plaque progression and promoting features of a stable plaque phenotype. However, its clinical utility is limited by dose-dependent thrombocytopenia due to BCL-xL inhibition in platelets and ambiguous results of studies on animal models [48,49]. HSP90 inhibitors represent another senolytic strategy by inducing cell cycle arrest and inhibiting the proliferation of VSMCs [50]. These molecules also increase Nrf2 activation, which reduces the pro-inflammatory activity of senescent cells [51].

Preclinical investigations, primarily conducted in hypercholesterolemic murine models such as ApoE^−/−^ and LDLR^−/−^ mice, have provided robust evidence supporting the anti-atherosclerotic efficacy of senolytic therapies. These studies consistently demonstrate that senolytic interventions selectively deplete senescent cells, thereby attenuating pathological plaque progression and significantly reducing overall atherosclerotic burden in rodents [46,47,50]. Treatment with the senolytic combination D+Q has been shown to improve vasomotor function in aged mice and to reduce aortic calcification and osteogenic signaling in hypercholesterolemic models [46]. Similarly, fisetin markedly attenuated atherosclerotic lesion formation in ApoE^−/−^ mice by suppressing oxidative stress and ferroptosis in aortic tissues [47]. Collectively, senolytic interventions improve endothelial function by enhancing nitric oxide bioavailability, reducing oxidative stress, and lowering circulating and vascular levels of pro-inflammatory cytokines [52]. These effects underscore the capacity of senolytics to eliminate senescent cells within cardiovascular tissues and to prevent or reverse disease progression [43]. Beyond atherosclerosis, senolytics have demonstrated therapeutic benefits across a range of cardiovascular pathologies, including attenuation of cardiac fibrosis and improvement in heart failure with reduced ejection fraction (HFrEF) [43]. In aged murine models of myocardial infarction, treatment with the senolytic agent ABT263 improved myocardial remodeling, enhanced diastolic function, and increased overall survival [53]. Furthermore, in murine heart failure models, senolytic therapy induced favorable cardiac remodeling characterized by reductions in fibrosis, hypertrophy, and inflammation, primarily through the apoptotic elimination of senescent cells [54] (see: Table 3).

### 5.2. Senomorphic Therapies in Atherosclerosis

Senomorphics are pharmacological agents that modulate the phenotype and functional activity of senescent cells without inducing their elimination. In contrast to senolytics, which selectively trigger senescent cell death, senomorphics attenuate or reprogram SASP, thereby mitigating its pro-inflammatory, pro-oxidative, and tissue-damaging effects [10,55]. Mechanistically, senomorphics act on multiple signaling pathways implicated in the regulation of cellular senescence. By modulating these pathways, senomorphics suppress SASP production while preserving senescent cell viability, distinguishing them from cytotoxic senotherapeutic strategies. This non-lethal modulation may be particularly advantageous in settings where the complete clearance of senescent cells is undesirable or impractical [56]. Importantly, this therapeutic approach is especially relevant in physiological and pathological contexts in which senescent cells exert beneficial functions, including wound healing, tissue repair, and tumor suppression. In such scenarios, the functional “silencing” of senescent cells through SASP modulation represents a safer and more nuanced alternative to their complete elimination [57].

Metformin, a widely prescribed antidiabetic agent, is among the most extensively studied senomorphic compounds. Its senomorphic activity is primarily mediated through activation of AMP-activated protein kinase (AMPK), inhibition of mTOR signaling, and suppression of NF-κB–dependent transcription. In vascular cells, metformin improves endothelial function, reduces ROS generation, and attenuates the secretion of SASP factors. Collectively, these effects confer protection against vascular inflammation and dysfunction, key processes underlying vascular aging and atherogenesis [58,59]. Rapamycin, a direct inhibitor of mTOR signaling, similarly exerts potent senomorphic effects by reducing SASP production and delaying vascular aging. Through enhancement of autophagy, suppression of anabolic signaling, and modulation of anti-atherogenic immune responses, rapamycin alleviates endothelial dysfunction and chronic vascular inflammation—central drivers of atherosclerotic disease progression [60,61]. Targeting downstream inflammatory signaling, inhibitors of the JAK/STAT pathway, such as ruxolitinib, directly suppress SASP-associated cytokine signaling. By limiting the expression of pro-inflammatory mediators, these agents improve vascular rheology and reduce immune cell recruitment to atherosclerotic plaques, thereby attenuating plaque inflammation and vascular remodeling [62,63].

Senomorphic therapies attenuate vascular inflammation, improve nitric oxide–mediated endothelial function, and promote plaque stabilization. By suppressing SASP–driven matrix degradation and immune activation, these agents slow atherosclerotic progression and may reduce cardiovascular risk. Importantly, senomorphics may be particularly suitable for elderly patients, in whom extensive senescent cell clearance could compromise tissue integrity and regenerative capacity [58,59]. In hyperlipidemic ApoE^−/−^ mice fed a high-fat diet, metformin did not reduce atherosclerotic plaque size; however, it inhibited phosphorylation of the AMPK/PGC-1α/TERT signaling axis, a pathway implicated in atherosclerotic plaque progression. Consistently, metformin attenuated atherosclerosis-associated phenotypes in both in vitro and in vivo models, supporting its modulatory rather than cytotoxic role in vascular disease [64]. Rapamycin has demonstrated anti-atherosclerotic effects through multiple mechanisms, including immune modulation and suppression of inflammatory signaling [60,61]. In aged LDLR^−/−^ mice, rapamycin treatment reduced total T-cell and macrophage accumulation in the aorta and spleen, promoted regulatory T-cell expansion, and decreased pro-atherogenic age-associated B cells within plaques, collectively indicating a shift toward anti-atherogenic immunity [61]. Complementary approaches using rapamycin-loaded biomimetic nanoparticles further reduced proliferating macrophages, dampened pro-inflammatory cytokine production, and favorably altered plaque morphology [65]. Targeting downstream inflammatory signaling, JAK/STAT inhibition has also shown therapeutic promise. In rabbit models of balloon injury–induced atherosclerosis, the JAK2 inhibitor ruxolitinib significantly reduced plaque burden and suppressed circulating pro-inflammatory cytokines, while increasing anti-inflammatory mediators [66]. Consistent with these findings, JAK/STAT inhibitors, including ruxolitinib and fedratinib, reduced leukocyte–endothelial adhesion and prothrombotic, proinflammatory vascular activation, highlighting their potential to mitigate both atherosclerosis and thrombosis [62] (see Table 4).

### 5.3. Next-Generation Senescence-Targeted Therapies

Immunosenolytic strategies represent a novel approach to selectively eliminate senescent cells using the immune system. In this context, chimeric antigen receptor (CAR) T cells engineered to recognize senescence-associated surface markers, such as urokinase-type plasminogen activator receptor (uPAR), have demonstrated the ability to selectively target and remove senescent cells. These immunotherapeutic strategies are further complemented by modulation of inflammatory pathways, particularly interleukin-1 (IL-1) signaling, as IL-1 inhibitors have been shown to effectively reduce inflammatory biomarkers and atherosclerotic burden [39]. In parallel, preclinical studies have shown that anti-oxidized low-density lipoprotein (OxLDL) CAR regulatory T cells (Tregs) significantly reduce macrophage foam-cell formation in vitro and markedly suppress atherosclerotic plaque development in vivo in immunocompetent murine models. By attenuating OxLDL-driven vascular inflammation and plaque accumulation, these engineered CAR Tregs underscore the potential of targeted immunosenolytic and immunomodulatory therapies as innovative treatment strategies for atherosclerosis [67].

Epigenetic dysregulation is increasingly recognized as a fundamental mechanism in the pathogenesis of cardiovascular diseases, particularly through its role in sustaining cellular senescence and SASP. Aberrations in DNA methylation and histone acetylation modulate key aging-related pathways, thereby promoting chronic inflammation and vascular dysfunction that characterize cardiovascular pathology [68]. In this context, the development of atherosclerosis has been strongly associated with environmentally acquired alterations in DNA methylation patterns, further underscoring the contribution of epigenetic mechanisms to disease progression [69]. Targeted epigenetic therapies, such as histone deacetylase (HDAC) inhibitors, have emerged as promising interventions that can regulate the expression of key inflammatory genes in senescent cells. By inducing chromatin remodeling, these agents may repress pro-inflammatory gene programs, offering the potential to reverse the senescent phenotype and restore cellular homeostasis [70]. In addition, evidence suggests that food-derived bioactive compounds can function as DNA methyltransferase (DNMT) inhibitors, thereby reshaping epigenetic landscapes relevant to cardiovascular disease [71]. Collectively, these findings indicate that modulation of epigenetic mechanisms through the administration of epigenetically active agents represents a viable and innovative therapeutic strategy for targeting cardiovascular disease at its molecular roots [72].

## 6. Discussion

### 6.1. Role of Cellular Senescence and SASP in Atherosclerosis

The mechanisms of cellular senescence and the senescence-associated secretory phenotype (SASP) significantly broaden our understanding of atherosclerosis development. The accumulation of senescent cells within the vascular wall—including endothelial cells, vascular smooth muscle cells and macrophages—leads to the loss of their physiological functions and to the secretion of pro-inflammatory cytokines, chemokines, growth factors and matrix-degrading enzymes. SASP components such as IL-6, IL-1β, IL-8, TNF-α and MMPs exacerbate chronic inflammation, disrupt tissue homeostasis and promote vascular remodelling, thereby contributing to atherosclerotic plaque initiation and progression. SASP acts both paracrinally, transmitting ageing signals to neighbouring cells, and endocrinally, affecting distant tissues, which further amplifies inflammatory processes and promotes plaque destabilisation [6,73,74].

A growing body of evidence highlights that not only the burden of senescent cells but also the qualitative composition of SASP critically shapes the vascular microenvironment, influencing disease severity and progression. Recent studies emphasise complex regulatory mechanisms of SASP, including epigenetic and metabolic pathways as well as external stimuli such as lipopolysaccharides, which can enhance both cellular senescence and SASP secretion [20,75,76]. At the same time, the biological complexity of SASP, its pleiotropic effects and its involvement in multiple signalling pathways highlight that senescence is not solely detrimental but may exert context-dependent functions, complicating attempts to therapeutically target this process [77].

### 6.2. Biological Consequences and Clinical Relevance

The progressive accumulation of senescent cells contributes to chronic, low-grade inflammation, commonly referred to as “inflammaging”, which represents a major risk factor for cardiovascular diseases, including atherosclerosis, hypertension and heart failure [42]. SASP-driven inflammation disrupts endothelial and smooth muscle cell function, increases vascular permeability and accelerates pathological vascular remodelling [6,78]. Furthermore, SASP impairs macrophage-mediated clearance of senescent and necrotic cells, thereby sustaining inflammatory signalling and promoting lesion expansion [79].

Clinically, an increased burden of senescent cells correlates with organ dysfunction, heightened plaque instability and an elevated risk of adverse cardiovascular events such as myocardial infarction and heart failure. However, experimental evidence suggests that the removal of senescent cells may paradoxically destabilise atherosclerotic plaques, impair cardiac function and, in some animal models, even increase mortality, raising important safety concerns regarding aggressive senolytic interventions [40,43]. These findings indicate that senescence may also play a structural or compensatory role in advanced lesions, underscoring the need for caution when translating senescence-targeting strategies into clinical practice. Many clinical observations remain preliminary and require confirmation in large, long-term cohorts [9,80].

### 6.3. Therapeutic Strategies Targeting Senescence

Therapeutic approaches aimed at eliminating senescent cells (senolytics) or modulating SASP without cell removal (senomorphics) have demonstrated promising results in preclinical studies and early-phase clinical trials. Senolytic agents such as dasatinib, quercetin, fisetin and navitoclax have been shown to reduce senescent cell burden, alleviate chronic inflammation, improve vascular and cardiac function and stabilise atherosclerotic plaques in experimental models [10,55]. Senomorphics, including inhibitors of the JAK/STAT signalling pathway (e.g., ruxolitinib), suppress SASP secretion and attenuate inflammation without directly inducing senescent cell death [6,42].

Despite these encouraging outcomes, the clinical implementation of senolytic therapies remains highly challenging. A fundamental limitation is the lack of specific and universal biomarkers that enable precise identification of all senescent cell subtypes, which compromises drug selectivity and increases the risk of off-target toxicity and adverse effects [10,43,55]. Moreover, some SASP components appear to fulfil protective or reparative roles, for instance in tissue regeneration and wound healing, suggesting that indiscriminate elimination of senescent cells or complete suppression of SASP may have detrimental systemic consequences [40,81].

A further limitation of senolytics is the potential removal of cells that maintain tissue integrity, whereas senomorphics mitigate inflammation but do not eliminate the underlying source of SASP. Importantly, most current evidence derives from preclinical models, while available clinical trial data remain scarce and insufficient to determine long-term efficacy, safety and optimal dosing strategies of senolytic agents [9,14]. Novel approaches, including targeted delivery systems and immunotherapies such as CAR-T cells directed against senescence-associated antigens, may improve selectivity and safety; however, robust clinical validation is still lacking [82,83].

### 6.4. Implications for Future Research

Future studies should prioritize the identification of reliable biomarkers of cellular senescence and SASP to enable early detection, patient stratification and monitoring of therapeutic responses [73,84]. Comprehensive evaluation of the safety and efficacy of senolytics and senomorphics is essential, particularly in the context of senescent cell heterogeneity and their interactions with the tissue microenvironment. A deeper understanding of the molecular mechanisms governing senescence induction, SASP regulation and systemic signalling is crucial to minimise unintended interference with essential physiological processes such as tissue repair, wound healing and cancer surveillance [6,81,85]. The development of advanced experimental models, including refined animal systems and single-cell technologies, may substantially accelerate progress in this field [10,43]. A comprehensive overview of cellular senescence, SASP roles, and therapeutic implications in atherosclerosis is provided in Table 5.

### 6.5. Relevance for Personalised Medicine

Elucidating the role of cellular senescence and SASP opens new avenues for personalised cardiovascular therapy, allowing the selection of senescence-targeting strategies tailored to individual patient profiles. Such approaches may slow atherosclerosis progression, improve cardiac function and enhance quality of life. Nevertheless, given the current limitations in therapeutic selectivity, safety and long-term clinical evidence, further extensive research is required to identify patient populations most likely to benefit from these interventions while minimising the risk of serious complications [73,78,85].

## 7. Conclusions

Cellular senescence and SASP drive chronic inflammation and vascular dysfunction central to atherosclerosis pathogenesis, with senescent endothelial cells, VSMCs, and macrophages promoting plaque formation, instability, and necrotic core expansion through proinflammatory cytokines, chemokines, and matrix metalloproteinases. Despite the well-described role of these mechanisms, it is still not entirely clear at which stages of atherosclerosis and for which senescent cells a therapy targeting senescent cells should be applied, and when its implementation might prove harmful.

Accumulation of senescent cells in arterial walls exacerbates inflammaging, endothelial permeability, VSMC phenotypic switching, and impaired efferocytosis, all hallmarks of atherosclerotic progression. Senolytic therapies, such as D+Q, fisetin, and navitoclax, selectively eliminate these cells in preclinical models, reducing plaque burden, oxidative stress, calcification, and improving endothelial function and plaque stability. Senomorphic agents like metformin, rapamycin, and JAK/STAT inhibitors suppress SASP without cell clearance, mitigating inflammation and vascular remodeling, while emerging immunosenolytics and epigenetic modulators offer targeted potential.

These mechanisms position senescence as an active driver—not bystander—of age-related CVDs, linking modifiable aging processes to major CVD risks like myocardial infarction and stroke. Targeting SASP and senescent cells via senolytics or senomorphics holds promise for preventing plaque destabilization and restoring vascular homeostasis, potentially complementing existing therapies with reduced toxicity.

Research must prioritize specific senescence biomarkers for patient stratification, long-term clinical trials to assess safety amid risks like plaque destabilization or loss of beneficial SASP functions, and refined strategies accounting for senescent cell heterogeneity and microenvironment interactions to enable personalized CVD interventions.

Given the limited knowledge on the safety and efficacy of these therapies in the context of atherosclerosis, further research on senescence markers, intervention selectivity, and the impact of SASP and senescent cells on the vascular microenvironment is necessary to develop safe and personalized therapeutic strategies.

## Figures and Tables

**Table 1:** Biology and mechanisms of cellular senescence.

Type/Mechanism of Senescence	Inducing Factors
Replicative senescence	Telomere shortening during repeated cell divisions
Telomere dysfunction	Accumulation of DNA damage at telomeres independent of telomere length
SA-β-galactosidase activity	Increased lysosomal content in senescent cells
Stress-induced premature senescence	Oxidative stress, radiation, toxins, oncogene overexpression
p53/p21 pathway–mediated senescence	DNA damage and cellular stress
p16^INK4A^/Rb pathway–mediated senescence	Persistent senescence-maintaining signals
Mitochondrial dysfunction in senescence	Decline in NAD^+^ and ATP, increased ROS

**Table 2:** Acute versus chronic SASP activity.

Feature	Acute SASP	Chronic SASP
Duration	Transient	Persistent
Primary function	Tissue repair and immune clearance	Sustained inflammation and degeneration
Immune response	Effective senescent cell removal	Immune evasion and chronic activation
Tissue outcome	Restoration of homeostasis	Inflammaging and disease progression

**Table 3:** Mechanisms and cardiovascular effects of senolytic therapies.

Senolytic Agent	Mechanism of Action	Cardiovascular Effects
Dasatinib + quercetin (D+Q)	Disruption of senescence-associated anti-apoptotic pathways (BCL-2/BCL-xL, PI3K/AKT, p53/p21), inducing selective apoptosis of senescent cells	Reduction in senescent cell burden and SASP, improved endothelial function, reduced aortic calcification, attenuation of atherosclerotic plaque progression
Fisetin	Inhibition of anti-apoptotic signaling in senescent cells, promoting senescent cell clearance	Suppression of oxidative stress and ferroptosis, marked reduction in atherosclerotic lesion formation
Navitoclax (ABT-263)	Pharmacological inhibition of BCL-2 and BCL-xL, triggering apoptosis of senescent cells	Restriction of plaque progression, promotion of plaque stability, improved myocardial remodeling and cardiac function in heart failure and post-infarction models
HSP90 inhibitors	Destabilization of pro-survival proteins in senescent cells and activation of Nrf2 signaling	Reduction in senescence-associated inflammation and inhibition of vascular smooth muscle cell proliferation

**Table 4:** Mechanisms and cardiovascular effects of senomorphic therapies.

Senomorphic Agent	Mechanism of Action	Cardiovascular Effects
Metformin	Activation of AMPK with downstream inhibition of mTOR and NF-κB, leading to suppression of SASP	Reduced vascular inflammation and oxidative stress, improved endothelial function
Rapamycin	Direct inhibition of mTOR signaling, suppression of SASP, and enhancement of autophagy	Attenuation of endothelial dysfunction and chronic vascular inflammation, promotion of plaque stabilization
JAK/STAT inhibitors (e.g., ruxolitinib)	Inhibition of SASP-associated cytokine signaling via blockade of the JAK/STAT pathway	Reduced plaque inflammation, decreased leukocyte recruitment, improved vascular remodeling
Epigenetic modulators (HDAC and DNMT inhibitors)	Epigenetic repression of pro-inflammatory senescence-associated gene expression	Suppression of chronic vascular inflammation and potential restoration of vascular homeostasis

**Table 5:** Summary of cellular senescence, SASP roles, and therapeutic implications in atherosclerosis.

Aspect	Key Points	References
Senescent cells in vessels	Accumulate in endothelium, smooth muscle cells, and macrophages; secrete pro-inflammatory SASP factors	[6,73,74]
SASP effects	Amplifies inflammation, destabilises plaques; effects are context-dependent	[6,40,73,74,81]
Senolytic therapies	Reduce senescent cell burden, inflammation; potential to improve vascular/cardiac function	[10,55]
Senomorphic therapies	Modulate SASP without cell removal; safer but do not eliminate source	[6,42]
Limitations and risks	Lack of biomarkers, potential plaque destabilisation, limited clinical data	[9,10,14,40,43,55]
Future directions	Identify biomarkers, optimise safety, enable personalised interventions	[6,10,43,81,84,85]

## Data Availability

No new data were created or analyzed in this study.

## Abbreviations

The following abbreviations are used in this manuscript:

CVDs	Cardiovascular diseases
VSMCs	Vascular smooth muscle cells
SASP	Senescence-associated secretory phenotype
ALT	Alternative lengthening of telomeres
SA-β-Gal	Senescence-associated β-galactosidase
SIPS	Stress-induced premature senescence
ECs	Senescent endothelial cells
H_2_O_2_	Hydrogen peroxide
OIS	Oncogene-induced senescence
ROS	Reactive oxygen species
OXPHOS	Oxidative phosphorylation
mTOR	Mechanistic target of rapamycin
SCAPs	Senescence-associated anti-apoptotic pathways
D+Q	Dasatinib and quercetin
HFrEF	Heart failure with reduced ejection fraction
AMPK	AMP-activated protein kinase
CAR	Chimeric antigen receptor
uPAR	Urokinase-type plasminogen activator receptor
IL-1	Interleukin-1
oxLDL	Oxidized low-density lipoprotein
Tregs	Regulatory T cells
CAR-Tregs	Chimeric antigen receptor regulatory T cells
HDAC	Histone deacetylase
DNMT	DNA methyltransferase
